# From Claims to Choices: How Health Information Shapes Consumer Decisions in the Functional Food Market

**DOI:** 10.3390/foods14040699

**Published:** 2025-02-18

**Authors:** Concetta Nazzaro, Anna Uliano, Marco Lerro, Marcello Stanco

**Affiliations:** Department of Law, Economics, Management and Quantitative Methods, University of Sannio, 82100 Benevento, Italy; auliano@unisannio.it (A.U.); mrlerro@unisannio.it (M.L.); mstanco@unisannio.it (M.S.)

**Keywords:** experimental auctions, WTP, consumer behaviour, health claims, health consciousness, BDM

## Abstract

The current study examines the impact of health claims on consumer preferences and willingness to pay (WTP) for functional snack bars, focusing on anti-inflammatory and antioxidant properties. Through an experimental auction involving 175 participants, this study investigates how providing clear information on product health benefits influences consumer interest and WTP while analysing the role of individual health consciousness (HC) in shaping these preferences. The results indicate that detailed health claims positively affect consumer WTP for functional snack bars compared to standard options. Although both anti-inflammatory and antioxidant claims attract consumer interest, no significant difference in WTP was observed between the two, suggesting similar perceived value for these distinct benefits. However, highly health-conscious consumers demonstrate a stronger preference and WTP for anti-inflammatory options, indicating that HC influences specific health claim valuation. These findings underscore the importance of effective health-related messaging in promoting functional foods and suggest that general health claims may resonate more broadly with consumers than specialised ones. This study’s results enhance the current knowledge on functional foods, especially snack bars, offering valuable insights for manufacturers aiming to implement targeted marketing strategies and public health initiatives focused on promoting healthier dietary choices.

## 1. Introduction

In recent decades, there has been a significant shift in consumers’ food choices, with extrinsic attributes such as brand, packaging, and labelling gaining increasing prominence. These factors have progressively approached, and in some cases exceeded, the importance traditionally attributed to intrinsic attributes, such as food taste and texture [1,2,3,4,5]. At the same time, rising health awareness has driven consumers to prioritise foods that align with specific health and lifestyle goals [6,7,8]. Consumers are increasingly focused on their foods’ nutritional content and are willing to pay a price premium for products perceived as healthier or offering additional benefits [9,10,11]. In this context, the information on food packaging may be crucial in shaping consumer perceptions and purchasing decisions. Studies have shown that consumers are more likely to perceive products as healthier and more appealing when information about ingredients, nutritional value, and potential health benefits are clearly and prominently displayed on the packaging [7,12,13].

This shift in consumer behaviour is also reflected in European policy initiatives aimed at promoting healthier eating habits. In response to the rising prevalence of non-communicable diseases (NCDs), such as obesity, diabetes, and cardiovascular diseases, European policies have increasingly focused on fostering nutritional awareness and encouraging the consumption of healthier food options [14]. The European Commission’s Farm to Fork Strategy, a core component of the European Green Deal, underscores the importance of providing clear and accessible information to consumers, enabling them to make informed dietary choices [15,16].

Increasing awareness of the health impacts of diet has shifted consumer preferences towards products that support overall well-being, such as functional foods. The role of information could be particularly relevant in this growing market, which offers a wide range of products. According to Diplock et al. [17] (p. 6), functional foods are defined as “foods for which if it is satisfactorily demonstrated to affect beneficially one or more target functions in the body, beyond adequate nutritional effects, in a way that is relevant to either improved state of health and well-being and/or reduction of risk of disease”.

Previous contributions demonstrated that information could shape consumer behaviour towards functional foods. Studies have shown that clear and concise labelling can significantly influence consumer willingness to try and repeatedly purchase functional products [18,19,20]. Moreover, consumers are more likely to pay a premium for functional foods when they perceive the health claims to be credible and the nutritional benefits to be well communicated [21,22]. However, while the generally positive impact of health information on consumer choices is well documented, less attention has been given to how consumers differentiate between various types of health claims within the functional foods category.

Given the wide range of functional foods, communicating specific health benefits effectively becomes crucial.

Anti-inflammatory and antioxidant properties are especially relevant in this context, as there is high consumer interest in these attributes due to the link between many health issues and inflammation or oxidative stress [23,24]. While consumers are generally aware of the potential advantages of functional foods, the differentiation between products, such as those claiming anti-inflammatory versus antioxidant properties, still needs to be explored. The extent to which these specific claims influence consumer preferences and their willingness to pay (WTP) represents a critical area of investigation. Furthermore, in today’s environment of information saturation, it is essential to determine whether providing additional detailed information about product functionalities aids in informed decision-making or merely adds to consumer confusion.

This study explores the role of detailed information within the functional food market, focusing on the differentiation between products with distinct claims. Specifically, this study investigates whether consumers perceive anti-inflammatory and antioxidant benefits differently and how information shapes such perceptions. By analysing consumers’ WTP, this study seeks to determine whether additional information enhances consumer awareness and preference for a particular product or if it leads to confusion due to information overload. To gain deeper insights into real-world consumer behaviour, this study goes beyond hypothetical scenarios through experimental auctions to elicit consumers’ preferences in a non-hypothetical setting.

The paper is structured as follows: Section 2 outlines this study’s background and articulates the research questions. Section 3 details the materials and methods employed, while Section 4 presents and discusses the results. Finally, Section 5 offers this study’s conclusions, implications, and limitations.

## 2. Background of This Study

Various factors influence consumers’ food choices, including price, brand, packaging, and socio-cultural context. Among these, intrinsic attributes like taste have traditionally been pivotal, often being the most critical element driving purchase decisions [25,26]. However, in recent years, extrinsic attributes have gained increasing prominence. Brand reputation, packaging design, and health-related information are crucial in shaping consumer preferences [27,28]. Health has emerged as a significant extrinsic factor, driving consumers toward foods perceived as beneficial for well-being, such as functional foods [29]. This shift has been driven by the rising prevalence of NCDs, underscoring the critical role of dietary choices in health maintenance, and this was further amplified during the COVID-19 pandemic as people became increasingly concerned about their overall health, leading to a heightened interest in maintaining a healthy and balanced diet [14,30,31,32,33]. Consequently, the global demand for functional foods has surged recently [34,35,36]. Initially popular in Japan, functional foods have rapidly expanded into industrialised countries, particularly in Europe, since the early 2000s, with notable growth in the UK, France, Germany, and the Netherlands [20,37]. According to Mark-Herbert [38], functional food encompasses products that provide specific health benefits beyond their normal nutritional functions. These products can vary in type (e.g., yoghurt, biscuits) and function, but they must deliver scientifically proven effects, such as delaying or preventing the onset of certain diseases, distinguishing them from curative products and medicines. Kotilainen et al. [39], Spence [40], and Sloan [41] classify functional foods into four categories: fortified products (foods fortified with added nutrients); enriched products (foods with the addition of new nutrients or ingredients not typically found in certain foods); modified products (foods from which a harmful ingredient has been removed or replaced with another with beneficial effects); and improved products (foods in which one of the ingredients has been naturally increased through special growing conditions, new feed formulations, or genetic modification).

The market for functional foods includes various items such as bakery products, breakfast foods, snacks, bars, dairy products, and baby foods. Dairy products lead global sales, followed by baby foods and bakery products. However, functional snack bars have emerged as one of the fastest-growing segments due to their convenience and alignment with the lifestyles of busy consumers [35,42,43]. On-the-go snacking is gaining popularity among children who look for healthier snack options and adults who prefer these products due to their hectic lifestyles. Functional snack bars are also easy to transport and often come in smart, eco-friendly packaging [42,43]. Several studies have investigated the factors influencing the purchase of functional foods. These factors can be broadly categorised into socio-demographic and psychological characteristics. According to Baker et al. [36], consumer attitudes are shaped by product characteristics, socio-demographic attributes, psychological factors, behavioural tendencies, and physical traits. Accordingly, Topolska et al. [44] found that age and gender significantly impact the likelihood of purchasing functional foods, with older individuals and women showing a greater propensity to prioritise health benefits over taste. Family health issues also heighten interest in these products.

Conversely, younger consumers often prioritise visual appeal, such as packaging imagery, over nutritional and health claims, although those focusing on healthy eating are more inclined toward functional foods [45,46]. Moreover, studies have found a link between health consciousness and the consumption of functional foods, suggesting a positive correlation between attention to health and the consumption of functional foods [47,48,49]. Notably, the presence of nutritional information about food and information concerning health benefits significantly increases consumers’ WTP for many functional products, including those containing upcycled ingredients (i.e., biscuits with defatted sunflower cake flour) [50], enriched foods [51,52], and organic and fortified foods [53].

Given the pivotal role of information in consumer decision-making, this study seeks to explore how specific health claims, particularly anti-inflammatory and antioxidant benefits, ingredients, and nutritional value, affect consumer perceptions and WTP for functional snack bars. The goal is to assess whether additional health information enhances consumer awareness and preference or contributes to information overload, diminishing clarity and purchase intent.

In light of these considerations, the present study addressed the following research questions:

Research Question 1: How does specific health information related to anti-inflammatory and antioxidant claims influence consumer preferences and WTP for functional snack bars?

Research Question 2: To what extent does the level of consumer health consciousness impact their preferences and WTP for functional snack bars with detailed health claims?

Focusing on functional snack bars, which are increasingly popular due to their convenience and perceived health benefits, this study aims to provide valuable insights into the interplay between health information, consumer awareness, and purchasing behaviour.

## 3. Materials and Methods

### 3.1. The Experimental Auction

The analysis was based on a non-hypothetical experimental auction [54,55] combined with a structured questionnaire aiming at detecting consumers’ sociodemographic characteristics, consumption habits, and psycho-graphic characteristics essentially related to individuals’ concerns towards their health, through the health consciousness (HC) scale, which has been validated in the literature [56]. The latter was evaluated by asking respondents to express their degree of agreement/disagreement on a 5-point semantic scale, ranging from 1 (i.e., strongly disagree) to 5 (i.e., strongly agree).

The experiment was held from July to December 2023 in a specialised and fully equipped experimental economics lab featuring multiple workstations, dedicated software, and video-conferencing capabilities to support in-person and online sessions.

Participants were recruited on a voluntary basis, following standard practice in behavioural economics experiments [57,58,59]. No quotas or stratified sampling techniques were applied, as this study examined individual decision-making in a controlled environment. The recruitment phase involved distributing flyers and posters and sending digital invitations via email to a group of individuals who had previously expressed their willingness to participate in market research studies. If interested, participants could reach the laboratory from Monday to Friday, from 9 am to 6 pm, after sending an email confirmation. Upon reaching the laboratory, participants were welcomed and led to their designated workstation, equipped with notebooks. Each participant was given a unique identification code to ensure anonymity. Before the beginning of the auction, participants were endowed with a EUR 15 voucher and provided written informed consent by signing a paper-based document. They were told not to communicate with each other during the experiment but encouraged to share their opinions freely. The procedure of the experiment was then explained to them. To ensure that participants understood the auction mechanism, a training round with different products other than snack bars was carried out [60]. Real money was used in the experiment to reduce the hypothetical bias.

Participants knew their bids could result in an actual purchase, encouraging them to reveal their WTP. This setup creates a more realistic decision-making environment compared to traditional hypothetical surveys. Although not all participants engage in a final transaction, the auction format effectively reduces the likelihood of overstating or understating preferences, as participants are incentivised to consider the consequences of their bids [61]. The experimental auction followed the Becker–DeGroot–Marschak mechanism (BDM), used in the economic behavioural field for WTP measurement [62,63,64]. Specifically, such an auction mechanism implies that each participant competes against a computer, rather than other participants, to win a specific product. The price offered may be different from the final price paid. Participants who bid above the price generated by the computer (randomly drawn from a distribution of reasonable prices usually defined by the marketer or researcher) will pay the computer’s price and win the product. This makes the BDM mechanism incentive-compatible, as each participant is encouraged to bid according to their true preferences [65].

According to Skuza et al. [66], the BDM auction offers several advantages over other experimental auction methods, as it is relatively easy to implement, can be conducted with just one participant at a time, and ensures that participants do not bid against each other, thus reducing bias since the final price is determined by a random draw rather than direct competition [67].

The experiment was ‘within-subject’ designed; it was repeated in the same format for each participant [68]. The auction used the full bidding technique; the individual bidder was asked about their WTP for a particular product. Precisely for this experiment, the objects of evaluation were three snack bars: an anti-inflammatory snack bar (A), not yet on the market; an antioxidant snack bar (B), not yet on the market; and a standard snack bar (C), already on the market. Each snack bar was evaluated in two rounds, lasting about 15 min. In Round 1, participants expressed their evaluation and WTP for each snack bar following a sensory test (visual, olfactory, and gustatory). In Round 2, participants expressed their evaluation and WTP following an informative message conveyed by reading the ingredients and nutritional characteristics of the product (Table 1). Specifically, each participant participated in six auctions, two for each snack bar. At the end of the auction, only one of the six auction rounds was randomly selected: each participant took home the bar only if they were a successful winner in the selected round.

### 3.2. Statystical Analysis

The data analysis followed a two-step approach. First, a descriptive analysis was conducted to summarise the socio-demographic, behavioural, and psycho-attitudinal characteristics of the sample. Continuous variables (e.g., age, household size) were reported as means and standard deviations, while categorical variables (e.g., gender, education, job, and family income) were presented as frequencies.

Second, to assess the statistical significance of differences in WTP values and consumer evaluations of the products, a *t*-test was employed [69]. Specifically, the *t*-test was used to compare the mean WTP values for different snack bars and rounds, as well as to evaluate differences in consumer assessments of the snack bars themselves.

In order to examine how HC affects WTP for the products, the sample was divided into two groups based on the mean HC score, resulting in two subgroups: a “high health-conscious” group and a “low health-conscious” group. After categorising the sample, an unpaired *t*-test was applied to examine differences in the WTP delta values (the difference in WTP between the initial and second rounds of the auction) between the two groups. This analysis focused on the impact of information exposure between rounds (information effect), specifically evaluating how health claims (anti-inflammatory or antioxidant) influenced WTP across the two health consciousness groups.

Prior to applying the test, the distribution of the WTP and evaluation scores was visually inspected using histograms, kernel density estimation, and normality plots. This preliminary graphical assessment suggested that both WTP and evaluation data followed an approximately normal distribution. To confirm this, the Shapiro–Wilk test was conducted, which did not reveal significant deviations from normality (*p* ≥ 0.05 for all WTP distributions). Based on these results, the *t*-test was deemed appropriate, as its assumptions were met.

All statistical analyses were conducted using Stata 17 (StataCorp LLC, College Station, TX, USA).

## 4. Results and Discussion

### 4.1. The Sample

A sample of 175 individuals above 18 years were involved in this study. Table 2 shows the socio-demographic characteristics of the sample. In particular, participants had an average age of 46 years (min. 22, max. 77, std. dev. 14.483) and were primarily female (64%). The average household size of the sample was about three members, and the degree of education was mainly high (only 1.71% of the sample did not attend high school, and about 33% had a master’s and/or a PhD). About 70% of the sample were employees, although 18% were students. The family income categorisation was based on a proxy question, following the approach used in similar studies [70], to avoid biases associated with direct income reporting. Most of the sample (65%) reported an average income, 29% declared an income level above the average, and 5.71% reported a below-average income.

### 4.2. Overview of Consumers’ Preferences Towards Snack Bars

About 60% of the sample consumed snack bars at least once in the past. Among the latter, only 6% consumed snack bars more than three times a week, while the others consumed them once a week (17%), two/three times a month (13%), once a month (48%), and less than once a month (16%). The preferred sales channels for purchasing snack bars were those belonging to large-scale distribution (supermarkets, hypermarkets, discount) (74%), while 12% used online shops and 9% used specialised stores. These results are in line with market data, which show that the market demand for functional snack bars is constantly growing, as such products can respond to the current needs of modern consumers, even though they have not yet consistently entered into the eating habits of individuals [35,42,43].

To understand consumers’ purchasing preferences, we asked participants to evaluate, on a Likert scale ranging from 1 (=not important at all) to 5 (=extremely important), the degree of importance of different attributes considered when purchasing a snack bar (Table 3). The results show that the most crucial attribute was related to other consumers’ recommendations, followed by the nutritional properties of the snack bar and its brand. At the same time, the least important attribute concerned the presence of offers and promotions.

Such results are partially new in the literature, as there are no relevant contributions highlight the role of other consumers’ recommendations in purchasing snack bars. In any case, it is widely believed that the opinions of relatives and friends can influence individuals’ food choices. This reliance on recommendations may stem from a lack of familiarity or personal knowledge about foods, prompting consumers to turn to trusted social sources as a heuristic or shortcut to inform their decisions [71,72].

On the other hand, as Baker et al. [36] highlighted in their review on consumer acceptance of functional foods, the presence of nutritional properties on functional food labels has been identified as a significant factor influencing consumer acceptance of such foods. The study of Díaz et al. [47] confirms such a result, highlighting that consumers tend to accept functional foods with claims related to nutritional properties.

Among other attributes, the brand also can play a crucial role in consumers’ choices. As Bimbo et al. [73] pointed out, brand knowledge affects individuals’ willingness to purchase functional yoghurts. Accordingly, the contribution of Mirosa et al. [74] shows that trust in the product brand represents a key factor capable of influencing consumers’ choice of functional foods. This tendency may stem from consumers’ habitual behaviours, as they often prefer familiar brands that signal reliable quality and consistency. Familiarity with a trusted brand reduces perceived risk, especially when health claims are involved, and simplifies decision-making [70].

As for price, consumers may perceive a higher price as an indication of the effectiveness and premium quality of functional foods, leading to increased purchase intention. On the other hand, a higher price may discourage some consumers from purchasing functional foods [36,74,75,76]. Therefore, it is essential for companies to carefully consider pricing strategies for their functional food products to ensure that they strike a balance between consumers’ perceived value and affordability.

Also, the energy value attribute shows a relatively high ranking compared to other attributes. This suggests that consumers assign considerable importance to the energy content of foods. These findings align with previous research by Mancini et al. [77], which found that consumers exhibiting more virtuous behaviour are particularly attentive to the energy value of foods. According to their study, such consumers also pay close attention to ingredients, including the quantity and type of salt. The importance attributed to energy value in our study reflects this ongoing trend of increased consumer awareness regarding the nutritional aspects of food products.

The relatively high standard deviations observed across all attributes in Table 3, ranging from 0.665 to 1.066, suggest significant variability in participants’ responses. This could be due to the diverse preferences and decision-making criteria among respondents. For example, attributes such as brand and advertising exhibited higher standard deviations (1.066 and 1.007, respectively), indicating that these factors may be more polarising or subject to individual interpretation. In contrast, offers and promotions had the lowest standard deviation (0.665), likely reflecting a more consistent valuation of this attribute across participants. The high standard deviations for most attributes reflect the heterogeneity in consumer preferences. This variability could stem from differing familiarity with the products, varying personal priorities (e.g., health-conscious consumers valuing nutritional properties more), or demographic differences within the sample. Large standard deviations may also indicate that the means are influenced by outliers, which could inflate the perceived importance of certain attributes. Such deviations highlight that while specific attributes may have higher mean importance, their relevance can vary widely across individuals.

### 4.3. Results of the Experimental Auction

At the end of the first and second rounds, we asked the participants to evaluate, through a nine-point Likert scale, their overall liking of the snack bars. Round 1 concerned only the sensory evaluation (visual, olfactory, and gustatory), while Round 2 also concerned the assessment of each snack bar’s properties, ingredients, and nutritional characteristics. Table 4 shows that the overall liking declared by participants for the anti-inflammatory and antioxidant snack bars increased significantly after showing information, while, regarding the standard snack bar, the overall liking decreased significantly.

As regards the WTP, in Round 1, participants expressed an average WTP for the anti-inflammatory and the antioxidant snack bars of EUR 0.642 and EUR 0.627, respectively (Table 5). On the other hand, the average WTP expressed for the standard snack bar was higher, and in particular, equal to EUR 1.218. In Round 2, after showing information concerning the snack bars’ properties, ingredients, and nutritional characteristics, the average WTP for the anti-inflammatory and the antioxidant snack bars increased significantly; the anti-inflammatory snack bar saw an increase of EUR 0.206, while the WTP for the antioxidant snack bar increased by EUR 0.172. Conversely, the participants’ WTP for the standard snack bar decreased significantly by EUR 0.170 in Round 2.

These findings underscore the strategic role of information in influencing modern consumers’ food purchasing choices, particularly about functional foods, for which the health component represents an intrinsic value. Such results align with those of Papoutsi et al. [78], who stated that clear information significantly affects the choice of functional snacks, as the latter allows for improvement in the perception of taste and increases the WTP. In fact, in our study, the overall liking of the snack bars increased after showing participants the information concerning the properties, ingredients, and nutritional value of the snack bars under analysis; therefore, the sensory characteristics of the products were also evaluated differently after understanding the beneficial properties related to their consumption. Also, the study of Topolska et al. [44] stated that the presence of claims concerning the beneficial effect on individuals’ health, and therefore the disease-preventative properties of functional foods, represent a vital source of consumer attraction.

Furthermore, we investigated differences between the WTP’s of the different snack bars for each round. The results were the same for Rounds 1 and 2 (Table 6). More precisely, the WTP for the standard snack bar differed significantly from both the WTPs for the anti-inflammatory and the antioxidant snack bars (A–C; B–C). On the contrary, no statistically significant difference existed between the WTPs participants declared for the functional snack bars (A–B).

Consequently, consumers perceived a significative difference between the standard snack bar and the functional snack bars, while they considered the anti-inflammatory and the antioxidant bars to have equal value within each round. Despite the distinct health claims, the lack of significant differentiation in WTP between the anti-inflammatory and antioxidant options implies that consumers may view both functional snack bars as comparable in value and efficacy.

### 4.4. The Effect of Health Consciousness on Consumers’ WTP

The sample had mean value of the HC scale of 4.068 (Table 7). The internal consistency of the scale was measured through Cronbach’s alpha. The value of 0.817 shows a good internal consistency for the HC scale, as Cronbach’s alpha value was greater than 0.70 [79].

We categorised participants based on the average HC score to analyse how respondents’ WTPs for the products under study varied with their HC. Specifically, the sample was split into two groups: a “low health-conscious” group (96 individuals), with HC levels below the mean value, and a “high health-conscious” group, with HC levels equal to or above the mean threshold (79 individuals).

An unpaired *t*-test was applied to examine differences between the WTP delta values for each consumer group, focusing on the impact of information exposure between rounds (info effect) (Table 8). This approach enabled us to determine if the change in WTP (induced by informational claims) varied significantly depending on health consciousness levels. The results indicate that only the WTP delta for the anti-inflammatory snack bar showed a statistically significant difference between the low and high health-conscious groups, suggesting that high health-conscious consumers may find anti-inflammatory information particularly compelling. Conversely, no statistically significant differences emerged in the WTP delta for the antioxidant and standard bars, implying that antioxidant information did not similarly impact consumer perception across health consciousness levels.

This result underscores the importance of message tailoring, as consumers with higher health consciousness might selectively respond to more specific health claims like anti-inflammatory benefits, while antioxidant information appears less impactful in this respect.

## 5. Conclusions

This study provides valuable insights into consumer preferences and WTP for functional snack bars, revealing several key trends in consumer behaviour. It was found that giving precise and detailed information about ingredients, nutritional value, and health benefits positively influenced consumer attitudes and increased their WTP for functional snack bars. Despite the distinct health claims of anti-inflammatory and antioxidant bars, no significant difference in WTP was observed, suggesting that consumers may not differentiate between these specific claims when purchasing. Additionally, the role of HC emerged as a critical factor in consumer preferences, especially for anti-inflammatory snack bars. High health-conscious consumers were willing to pay significantly more for anti-inflammatory snack bars than low health-conscious consumers. This indicates that health-conscious consumers are more sensitive to health-related information and value products that explicitly support their health goals. However, the WTP for antioxidant snack bars did not vary significantly across the health consciousness groups, indicating that the impact of specific health claims may be more pronounced for certain consumers rather than being universally influential.

These findings have important implications for both manufacturers and marketers of functional foods. Marketers may benefit from emphasising general functional benefits (health and wellness) rather than focusing exclusively on specific health claims, such as anti-inflammatory and antioxidant effects. This could allow for simplified messaging highlighting overall health benefits, potentially appealing to a broader audience without differentiating between specific functions. Furthermore, understanding the importance of health consciousness will enable brands to better segment and target their audiences, tailoring their marketing efforts to appeal to consumers who prioritise specific health benefits in their purchasing decisions.

Public health initiatives could also benefit from these findings, particularly in the context of promoting healthier eating habits. Educational campaigns that focus on increasing consumer awareness about the benefits of functional foods and the importance of clear nutritional information could support healthier food choices across diverse consumer groups. Such initiatives could be further strengthened through partnerships between health organisations and food manufacturers to develop clear, informative packaging that communicates the functional benefits of snack products.

While this study provides important insights, it is limited by the sample size, which may only partially represent the broader population. Furthermore, the experimental auction methodology, involving a non-representative sample, could introduce biases such as selection bias, where participants may be more engaged or motivated than the general public, the endowment effect, where participants overvalue the auctioned goods simply because they are part of the experiment, and the social desirability bias, where individuals may inflate their WTP to appear more altruistic. Future research could explore the long-term effects of information provision, the influence of specific product attributes on WTP, and the impact of different communication channels on consumer perceptions.

## Figures and Tables

**Table 1:** Information provided to participants at Round 2.

A	B	C
A is a snack bar with anti-inflammatory properties.	B is a snack bar with antioxidant properties.	C is a standard snack bar.
Ingredients: Concentrated pineapple pulp (20%) (pineapple pulp, gelling agent: pectin), concentrated orange pulp (20%) (orange pulp, gelling agent: pectin), millefiori honey, chestnut flour, rice crispies (rice flour, sugar, salt), blown corn, dark chocolate (cocoa paste, brown sugar, cocoa butter, emulsifier: soy lecithin), bitter cocoa powder.	Ingredients: Pomegranate puree from concentrate (40%) (pomegranate pulp, gelling agent: pectin, stabilizing agent: inulin), millefiori honey, chestnut flour, puffed corn, rice crispies (rice flour, sugar, salt), dark chocolate (cocoa paste, cane sugar, cocoa butter, emulsifier: soy lecithin), bitter cocoa powder, coconut flakes.	Ingredients: Glucose-fructose syrup, oat flakes (gluten-free) (16%), loading agent: polydextrose, hazelnuts (12.4%), apricots (8%), raisins (6%), corn flakes (corn, sugar, salt) (5.5%), sunflower oil, dehydrated apple (3.6%), whole grain rice puff (2.5%), rice flakes (rice flour, corn meal, sugar, salt) (2.3%), acacia fibre, vitamin mix (maltodextrin, L-ascorbic acid, D-alpha-tacoferil acetate, nicotinamide, pyridoxine hydrochloride, thiamine hydrochloride), emulsifier: lecithin, natural aroma.
Nutritional values, per 100 g: Energy 1160 kj (290 kcal) Fats: 2.9 g (of which saturated 1.1 g) Carbohydrates: 61 g (of which sugars: 32 g) Dietary fibre: 2.2 g Protein: 3.9 g Salt: 0.18 g	Nutritional values, per 100 g: Energy: 1226 kj (301 kcal) Fats: 5 g (of which saturated: 2.7 g) Carbohydrates: 58 g (of which sugars: 25 g) Dietary fibre: 4.3 g Proteins: 3.8 g Salt: 0.15 g	Nutritional values, per 100 g: Energy: 1626 kj (389 kcal) Fats: 15 g (of which saturated: 1.3 g) Carbohydrates: 52 g (of which sugars: 24 g) Dietary fibre: 15 g Proteins: 5.5 g Salt: 0.15 g

**Table 2:** Samples’ socio-demographic characteristics.

Variable	Categories	Mean	Freq. (%)	Std. Dev.	Min.	Max.
Age		46.851		14.483	22	77
Gender	Male		36.0			
	Female		64.0			
Household size		2.811		1.283	1	8
Education	Primary school		0			
	Secondary school		1.71			
	High school		27.43			
	University degree		37.71			
	Master’s and/or PhD		33.14			
Job	Employee		70.29			
	Freelancer		4.0			
	Pensioner		6.86			
	Student		18.29			
	Other (unemployed, housewife/household, etc.)		0.57			
Family income	Below-average income		5.71			
	Average income		65.14			
	Above-average income		29.14			

**Table 3:** Attributes ordered by relevance.

Variable	Mean	Std. Dev.
Recommendations	3.962	0.95
Nutritional properties	3.790	0.997
Brand	3.476	1.066
Price	3.352	0.940
Energy value (kcal)	3.276	0.966
Advertising	2.581	1.007
Ingredients	2.581	0.978
Offers and promotions	1.648	0.665

**Table 4:** Participants’ overall liking of the snack bars.

Round	A	B	C
Round 1 (sensory)	4.503 (±2.133)	4.217 (±2.089)	7.0 (±1.360)
Round 2 (info + sensory)	5.651 (±1.974)	5.4 (±2.059)	6.366 (±1.573)
Round 2–Round 1 (info effect)	1.148 *** (±1.516)	1.183 *** (±1.619)	−0.634 *** (±1.536)

*** *p* < 0.01, ** *p* < 0.05, * *p* < 0.1.

**Table 5:** Average WTP for each snack bar (EUR).

Round	A	B	C
Round 1 (sensory)	0.642 (±0.551)	0.627 (±0.565)	1.218 (±0.696)
Round 2 (info + sensory)	0.848 (±0.642)	0.8 (±0.633)	1.048 (±0.670)
Round 2–Round 1 (info effect)	0.206 *** (±0.442)	0.173 *** (±0.368)	−0.170 *** (±0.389)

*** *p* < 0.01, ** *p* < 0.05, * *p* < 0.1.

**Table 6:** WTP difference among snack bars (EUR).

Round	A–B	A–C	B–C
Round 1	0.015 (±0.460)	−0.576 *** (±0.677)	−0.591 *** (0.655)
Round 2	0.048 (±0.522)	−0.2 *** (±0.690)	−0.248 *** (±0.698)

*** *p* < 0.01, ** *p* < 0.05, * *p* < 0.1.

**Table 7:** Descriptive statistics for HC scale.

Variable	Item	Mean	Std. Dev.
HC_1	I reflect on my health a lot	4.143	0.741
HC_2	I am very self-conscious about my health	4.234	0.717
HC_3	I am alert to changes in my health	4.177	0.725
HC_4	I am constantly examining my health	3.857	0.778
HC_5	I’m usually aware of my health	4.023	0.75
HC_6	I notice how I feel physically as I go through the day	3.971	0.812
HC_mean		4.068	0.545

**Table 8:** Consumers’ WTP delta (info effect) by different levels of health consciousness.

Variable	Low Health-Conscious		High Health-Conscious		Diff.
	N	Mean	N	Mean	Δmean
WTP_A_delta	96	0.151 (±0.433)	79	0.273 (±0.446)	0.122 ** (±0.067)
WTP_B_delta	96	0.154 (±0.395)	79	0.194 (±0.334)	0.040 (±0.055)
WTP_C_delta	96	−0.154 (±0.338)	79	−0.191 (±0.444)	−0.037 (±0.061)

*** *p* < 0.01, ** *p* < 0.05, * *p* < 0.1.

## Data Availability

Dataset available on request from the authors.

## Abbreviations

The following abbreviations are used in this manuscript:

WTP	Willingness to pay
HC	Health consciousness
BDM	Becker–DeGroot–Marschak
